# Using a Virtual Reality Tool to Provide Primary Prevention Training in the Construction Field Following a Periodic Medical Visit: Cross-Sectional Study

**DOI:** 10.2196/49218

**Published:** 2024-03-15

**Authors:** Sylvain Chamot, Isabelle Mahieu, Marion Delzard, Léa Leroy, Gwen Marhic, Maxime Gignon

**Affiliations:** 1 Regional Center for Occupational and Environmental Pathologies of Hauts-de-France Amiens University Hospital Amiens France; 2 Péritox (UMR_I 01) UPJV/INERIS Amiens France; 3 Department of General Medicine Amiens University Hospital Amiens France; 4 CRP-CPO, UR UPJV 7273 Université de Picardie Jules Verne Amiens France; 5 Education and Health Practices Laboratory UR3412 Sorbonne Paris Cité University of Sorbonne Paris Nord Bobigny France; 6 Department of Prevention, Risks, Medical Information and Epidemiology Amiens-Picardie University Hospital Amiens France

**Keywords:** virtual reality, virtual training tool, prevention, occupational medicine, construction

## Abstract

**Background:**

The construction field is highly concerned with the risk of work-related accidents, and training employees is difficult due to their small numbers in most companies.

**Objective:**

This study aimed to study the impact of a virtual reality (VR) training tool following a periodic occupational health medical visit on the feeling of personal effectiveness in preventing occupational risks related to co-activity on a construction site.

**Methods:**

We conducted a cross-sectional study with employees who had a periodic medical visit between April 1, 2022, and October 13, 2022, in a French occupational health service specializing in the construction field (Services Médicaux Interentreprises Bâtiment Travaux Publics [SMIBTP]). The employees were divided into 2 groups according to the training received: a medical visit alone or coupled with a session with a VR tool. We compared the scores for a “feeling of self-efficacy in occupational risk prevention” using the Fisher exact test.

**Results:**

Of the 588 employees included, 210 had a medical visit alone, and 378 had a medical visit coupled with VR training. Training with the VR tool was associated with an increased “feeling of self-efficacy in occupational risk prevention.” The employees who benefited from the training reported a willingness to apply the advice given on prevention to a greater extent than those who did not, and they believed that risks on the worksite could be reduced using this tool.

**Conclusions:**

Using VR training as a complement to periodic medical visits in an occupational health service improves the feeling of personal effectiveness in occupational risk prevention at the end of the training. If this trend is confirmed over a longer period of time, it could be an easily accessible prevention lever for employees in the future.

## Introduction

### Overview

The building and construction field is one of the most hazardous occupations in France. The main risks identified are the risk of road accidents, chemical risks, and musculoskeletal disorders, as well as risks related to the work environment and working with equipment. There are numerous work-related accidents, both fatal and nonfatal, with a major medicosocial impact on the individual and the community (long and costly medical care, prolonged absence from work negatively affecting the employer) [1]. In France, it is the leading field in terms of the frequency of work-related accidents, and the prevention of occupational risks remains difficult to achieve [2].

These difficulties are specifically related to collective prevention, which needs to be applied to a wide variety of tasks, sometimes in varying conditions and subject to change. Co-activities may be performed with employees from other companies in locations regularly situated very far from the company's head office. Small and medium-sized construction companies are the most affected by the lack of accessible risk prevention [3]. They also represent 99.8% of the companies and 45.7% of the jobs in France [4].

### Health Monitoring of Employees in France

In France, all employees must undergo a periodic medical visit (at least every 5 years) in an occupational health services center. The main mission is to avoid any alteration in the employees' health due to their work. Thus, during these visits, employees receive advice to prevent occupational risks [5].

The Services Médicaux Interentreprises Bâtiment Travaux Publics (SMIBTP) is an occupational health service in charge of medical visits in the field of building and construction activity. In 2018, the SMIBTP monitored 2001 companies and 15,176 employees, mostly working in small and medium-sized companies. These companies work on a large number of sites throughout northern France. Since February 2022, the SMIBTP has been experimenting with a virtual reality (VR) training tool to train employees in the primary prevention of occupational risks related to co-activities on construction sites at one of its 2 consultation centers. The VR training is always preceded by a medical visit.

### Virtual Reality

VR is a computer technology that involves real-time simulation and interaction through visual and auditory sensorial channels [6]. Computer-based 3D environments provide sensory information in a form similar to that received from the real world. VR allows individuals to experience and interact with or within environments with enhanced feedback [7-9]. To do this, users are required to be equipped with a VR headset that uses the principle of a stereoscopic 3D display connected to a computer interface to enable reproduction of the sensation of interaction with the artificial environment. The SMIBTP is the first occupational health service in France to have used a VR tool to provide additional prevention training for employees undergoing periodic medical visits. The goal of the SMIBTP is to provide employees with additional training in occupational risk prevention, with the aim of reducing the risk of accidents on site.

### Objectives

No study has yet been conducted on periodic medical visits in an occupational health service coupled with a VR educational tool. A study in Finland compared VR with lecture-based safety training and found that the feeling of personal effectiveness in occupational risk prevention was increased by VR at the end of the training [10]. On a more general note, a review of the literature was carried out in 2023 on VR training and its impact on prevention, focusing in particular on the construction sector and its risks, highlighting that, although there appeared to be a positive impact, there was a lack of experimental studies in this field [11]. This was also highlighted in a meta-analysis published in 2023 [12]. However, it's important to keep in mind that these reviews pool together studies with different methods. Some studies are based on immersive technologies such as head-mounted displays, which rely on a computer connection [13], and mobile VR, which relies on the use of a smartphone [14]. Others have used less immersive techniques such as the Cave Automatic Virtual Environment, which involves virtual reality spaces where the walls, floor, and ceiling act as huge projection surfaces [15]. For the same method, the tools may vary (eg, headset brand), and above all, the context of the serious game may be very different (eg, risk prevention specific to certain trades vs risk prevention linked to co-activity on construction sites here).

The main objective of our study was to determine whether VR training had an impact on the feeling of self-efficacy in occupational risk prevention compared with a medical visit alone.

As a secondary objective, we wanted to know how the employees rated this additional VR training compared with the medical visit alone.

## Methods

### Design

This cross-sectional study included employees coming for a periodic medical visit to the SMIBTP who presented between April 1, 2022, and October 13, 2022, at one of the 2 centers.

The employees received 2 types of prevention training depending on the center in which they were examined. The employees in the first group had a medical visit coupled with VR (MV+VR group) training at the end of their periodic medical visit (Site A). The employees in the second group (Site B) had a medical visit alone (MV group).

Only employees performing manual work on construction sites were included in the study (engineers or secretaries were not included). In addition, in the VR group, only employees who completed the entire training (eg, no interruption due to motion sickness) were included.

The only exclusion criteria were an employee's past or present refusal of personal data collection and an insufficient knowledge of French.

### Periodic Medical Visit

The role of occupational health services in France is to prevent any damage to workers' health caused by their work. The periodic medical visit is a preventive training tool used to this end. During these visits, workers can receive individual advice adapted to the workstation they occupy within their company. This involves oral advice, for example, on wearing personal protective equipment or using collective protective equipment. It may also, for example, involve a physical examination to assess the way employees bend over to pick up items from the floor. Finally, it may involve the delivery of paper documentation specific to the risks and workstations concerned.

### Virtual Reality Tool

The VR training tool used by the SMIBTP is a serious game entitled SRC-Bâti VR (ViRtual Création), which aims to improve the occupational risk prevention skills of construction workers using VR digital simulation. SRC-Bâti VR emphasizes the co-activity aspect of construction sites and therefore the interaction between employees with very different workstations. Relative to a typical medical visit, it is less theoretical and more closely approaches real work, which is expected to have a positive impact in terms of prevention [16]. ViRtual Création was created in 2018 to develop software as an educational tool to improve worker safety.

The training sessions lasted between 7 minutes and 10 minutes, and a technician was present to equip the employee and explain how the device works. The training took place in a dedicated area of more than 10 m². The technician did not interfere during the training, except, for example, to prevent the employee from colliding with the equipment in the room.

During the training, the employees moved freely on a construction site. Workstations were clearly identified by markers. When the employee went to a workstation, a multiple choice question appeared about an accident risk at the workstation. If the employee did not answer correctly, the accident occurred, and a correction was provided. When an accident occurred, the employee's senses were stimulated to raise awareness of the risk. Workstations at which there was a risk of falling made a strong impression, as the impression of falling was real, as were situations in which there was a risk of being crushed. SRC-Bâti VR therefore offered a realistic simulation that served to teach skills in the prevention of occupational hazards linked to on-site co-activity. This realistic aspect gave a dimension of play to the VR simulation, with employees positively reacting to these virtual accidents, sometimes providing them with a simulation of what would happen (employees were never evaluated on their performance in the questions, which served only as an introductive teaching aid).

**Figure 1:**
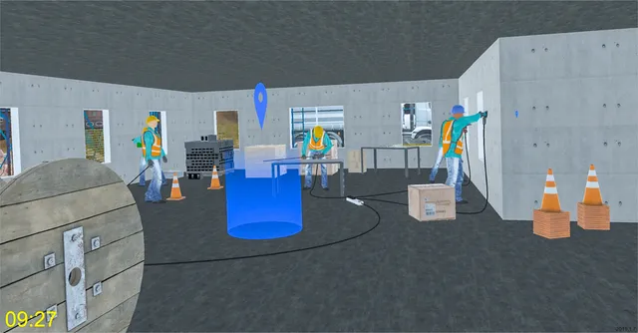
Representation of a workstation accessible by the employee in the Bâti VR serious game.

**Figure 2:**
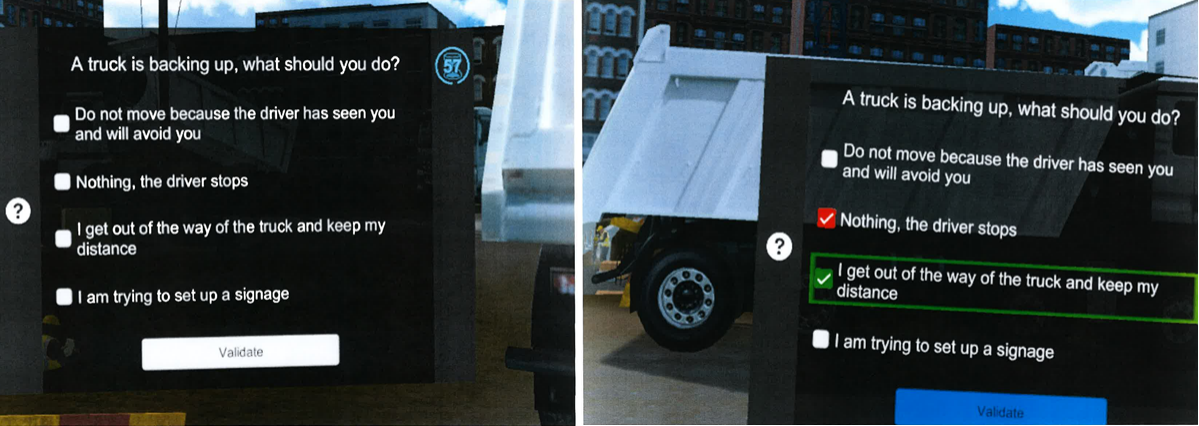
Example of a workstation in the Bâti VR serious game in which an employee is at risk of being run over by a truck.

Depending on the employee's profession, 2 types of VR training were possible: one focused more on road work, and the other focused on building construction. Of the 20 possible workstations, 7 were randomly presented during the training, and 1 had to be present (possible workstations are shown in Table 1). No other customization was implemented in addition to the basic tool. Employees moved around the site by teleporting from one workstation to another over short distances, rather than gliding along, using joysticks. Although the training is short, the involvement of participants and interactivity and immersion offered by VR distinguish it from a simple paper questionnaire with the same questions (certainly greater involvement). The risks addressed were representative of the major risks on a construction site. Figure 1 illustrates how an employee is notified of a workstation, and an example of a workstation is shown in Figure 2. Figure 2 shows, on the left, the initial risky situation in which a truck backs up toward the employee in training and, on the right, the correction involves the employee moving away from the truck (green proposal). The red proposal indicates that the employee made the wrong choice before the correction and was run over by the truck. Demos can be viewed online [17]. The headset was a VIVE Focus 3 because, at the time the training was set up in 2021, it was the headset recommended by ViRtual Création and ViRtual Création was, at the time, the only French company identified by the SMIBTP that offered ready-made training material for building construction and road works. Since then, another solution dedicated to on-site risk prevention has appeared in France: VIRTUAL CONSTRUCT (Mimbus).

**Table 1:** Possible workstations depending on the training scenario in the Bâti VR serious game.

Scenario	Workstations common to both scenarios	Scenario-specific workstations
Road work	Putting on personal protective equipment upon arrival on site^a^ Handling an unstable catwalk Putting safety caps on the ends of iron bars Waiting for trench walls to be reinforced to avoid being buried and limit machine traffic nearby How to limit the risks associated with the vibrations from a jackhammer How to deal with a truck backing up on a worksite In front of an area cluttered with equipment, clearing a passageway without the possibility of falling objects before carrying out work in this space In front of a colleague passing close to a load-lifting machine, informing the driver of the presence of the colleague to avoid any accidents Using safety barriers when passing near holes in the ground	Not driving past construction machinery but going around it by following the markings on studs Using appropriate personal protective equipment when operating a circular saw Using available handling equipment to carry loads instead of carrying them yourself Replacing defective site signage When laying asphalt on the road, wearing gloves, long sleeves, and pants for protection Not working on a running construction machine engine Using antipollution kits in the event of an accidental chemical spill on site Bypassing work areas and following safe paths when moving around the site When a construction machine reaches a buried network, stopping the machine and continuing work by hand Warning a truck driver if he is going to hit a power cable when reversing When climbing into a construction machine, always maintaining 3 points of support
Building construction	Putting on personal protective equipment upon arrival on site^a^ Handling an unstable catwalk Putting safety caps on the ends of iron bars Waiting for trench walls to be reinforced to avoid being buried and limit machine traffic nearby How to limit the risks associated with the vibrations from a jackhammer How to deal with a truck backing up on a worksite In front of an area cluttered with equipment, clearing a passageway without the possibility of falling objects before carrying out work in this space In front of a colleague passing close to a load-lifting machine, informing the driver of the presence of the colleague to avoid any accidents Using safety barriers when passing near holes in the ground	How to avoid injury when carrying a heavy load On scaffolding, limiting the risk of accidents by avoiding the presence of people working on several levels Ventilating and vacuuming when using a sander Before working on a pressurized water pipe, turning off the water supply completely When using electrically-powered machines, never repairing the machine or its connections yourself Using appropriate personal protective equipment when working near a colleague using a grinder Disposing of rags soaked in chemical products after use Reducing noise exposure by enclosing the compressor in dedicated rooms Wearing appropriate gloves when welding Alerting the colleague in charge of any abnormalities in load-bearing equipment Using rolling scaffolding for occasional work at height

^a^Workstation mandatory for all training sessions.

### Ethical Considerations

As this was a cross-sectional study evaluating current practice in the use of virtual reality, it did not require review by an institutional review board. Virtual reality was used independently of the study, with only an anonymous virtual reality evaluation questionnaire added by our teams. The study was carried out in compliance with good data protection practice, with the agreement of the data protection officer of the Université de Picardie Jules Verne. Our study was not funded by ViRtual Création and we did not collaborate with the company in the conduct of the study.

### Data Collection and Variables

We collected data using a questionnaire built using the LIMESUVEY tool provided by the University of Picardie Jules Verne. The questionnaire was completed directly following the intervention (site A) or after the medical visit (site B). The data collected were demographics (age, gender, size of the company in which the employee worked), type of medical visit (with or without VR), and questions related to the feeling of self-efficacy and their rating of the training using 5-point Likert scales. These questions have not been validated and were defined by the authors. The questionnaire we used, based on the LIMESUVEY tool, also did not undergo a prior validation study. It was, however, partially based on the model for self-efficacy questions by Kirkpatrick and Kirkpatrick [18], which is a training evaluation method based on 4 levels: reaction, learning, behavior, and results. It enables assessment of the effectiveness of a training program at different levels, from participant reactions to concrete results for the employer. This model is widely used in training and human resources development to measure the impact of training programs. We only studied reactions, as our study design did not allow for employees to be contacted at a later date. The immediate reaction was assessed by the statement “I feel more effective in prevention.” We wished to address the question of what employees felt they could apply in practice just after their training, in particular regarding on-site co-activity, using the following 2 statements: “I am ready to apply these prevention rules” and “I think that these prevention rules can reduce the risks with regard to other colleagues on the site.” The other 2 questions were aimed at evaluating the training received in itself: “My visit to the SMIBTP was worth it” and “I learned about prevention.”

To explore gender, we asked employees to indicate whether they defined themselves as male or female.

### Statistical Analysis

Employees were divided into 2 groups based on the 2 types of prevention training (MV *vs* MV + VR). The primary endpoint was a difference (as a percentage) between the responses of the 2 groups for each item (on our Likert scale) on questions relating to “feelings of self-efficacy in the prevention of occupational risks.” The secondary endpoint was the difference (as a percentage) between the responses of the 2 groups to questions relating to the rating of the training. Responses measured on the Likert scales were not transformed into a quantitative variable, to not distort the nature of this mode of questioning.

Baseline demographics and clinical characteristics are expressed as means (SDs) or medians (IQRs) for numerical variables and frequencies (percentages) for categorical variables. Between-group comparisons were performed using the Mann-Whitney *U* test (for age) and Fisher exact test for categorical variables (size of the company and gender). The chi-squared test could not be used because the number of participants for certain response modalities was <5. The Fisher exact test was used to assess the association between the type of prevention training and the primary and secondary endpoints. A *P* value of .05 was considered significant for all tests.

All statistical tests were performed using R software (version 4.0.0, R Core Team, R Foundation for Statistical Computing). The data and R scripts are available on MENDELEY [19].

## Results

During the study period (April 1, 2022, to October 13, 2022), 588 employees were recruited.

The baseline participant characteristics by type of prevention training are summarized in Table 2. The study population was predominantly male (571/588, 97.1%). The mean age was 33.15 (SD 12.1) years. By comparison, in 2019, men represented 87.89% of employees in the construction industry in France, and the mean age was 42 years [20,21]. There was not a statistically significant difference between the 2 groups in terms of gender, but there were statistically significant differences for age and company size. Of the 588 employees, there were 210 employees (35.7%) who had the medical visit alone (MV group) and 378 employees (64.3%) who had the medical visit coupled with VR training (MV+VR group). There were no missing data.

The results for the “feeling of self-efficacy in occupational risk prevention” are shown in Table 3. The MV+VR group had a greater feeling of self-efficacy in prevention than the MV group. For each question, there was a statistically significant difference at the 5% risk level, indicating that the MV+VR group felt more effective in prevention in general and, more specifically, in co-activity on worksites and would be more inclined to apply the prevention rules learned during their visit to the occupational health service.

The results of the ratings of the interventions received by the 2 groups are shown in Table 4. Employees in the MV+VR group found the intervention to be more useful and to provide more knowledge in terms of prevention than those in the MV group.

**Table 2:** Study population characteristics by type of prevention training at the Services Médicaux Interentreprises Bâtiment Travaux Publics (SMIBTP).

Characteristic			Overall (n=588)		MV^a^ (n=210)		MV+VR^b^ (n=378)		*P* value	
Age (years), median (IQR)			32 (23-42)		38 (28-47.75)		29 (21-37)		<.001	
Male, n (%)			571 (97.1)		201 (95.7)		370 (97.8)		.19	
**Size of the company**										.01
	1-10 employees	214 (36.4)		60 (28.6)		154 (40.7)				
	11-49 employees	224 (38.1)		91 (43.3)		133 (35.2)				
	50-299 employees	129 (21.9)		53 (25.2)		76 (20.1)				
	≥300 employees	21 (3.6)		6 (2.9)		15 (4)				

^a^MV: medical visit.

^b^MV+VR: medical visit coupled with virtual reality training.

**Table 3:** Distribution of answers relating to the “feeling of self-efficacy” statements.

Questions		Responses, n (%)						*P* value	
		Strongly agree	Agree	Neutral	Disagree	Strongly disagree			
**I feel more effective in prevention.**									.002
	MV^a^ (n=210)	77 (36.7)	87 (41.4)	32 (15.2)	3 (1.4)	11 (5.2)			
	MV+VR^b^ (n=378)	139 (36.8)	197 (52.1)	36 (9.5)	2 (0.5)	4 (1.1)			
**I am ready to apply these prevention rules.**									<.001
	MV (n=210)	118 (56.2)	70 (33.3)	16 (7.6)	2 (1)	4 (1.9)			
	MV+VR (n=378)	277 (73.3)	96 (25.4)	5 (1.3)	0	0			
**I think that these prevention rules can reduce the risks with regard to other colleagues on the site.**									<.001
	MV (n=210)	117 (55.7)	66 (31.4)	20 (9.5)	2 (1)	5 (2.4)			
	MV+VR (n=378)	282 (74.6)	90 (23.8)	5 (1.3)	0	1 (0.3)			

^a^MV: medical visit.

^b^MV+VR: medical visit coupled with virtual reality training.

**Table 4:** Distribution of answers relating to the evaluation of the training.

Statements		Responses, n (%)						*P* value
		Strongly agree	Agree	Neutral	Disagree	Strongly disagree		
**My visit to SMIBTP^a^ was worth it.**								.002
	MV^b^ (n=210)	129 (61.4)	65 (31)	13 (6.2)	1 (0.5)	2 (1)		
	MV+VR^c^ (n=378)	268 (70.9)	104 (27.5)	6 (1.6)	0	0		
**I learned about prevention.**								<.001
	MV (n=210)	75 (35.7)	93 (44.3)	19 (9)	4 (2)	19 (9)		
	MV+VR (n=378)	186 (49.2)	158 (41.8)	25 (6.6)	3 (0.8)	6 (1.6)		

^a^SMIBTP: Services Médicaux Interentreprises Bâtiment Travaux Publics.

^b^MV: medical visit.

^c^MV+VR: medical visit coupled with virtual reality training.

## Discussion

### Principal Findings

The results of our study show that the use of a VR training tool at the end of periodic occupational medical visits had an impact on the feeling of self-efficacy in terms of occupational risk prevention in the construction field. This is an important finding, suggesting that the use of VR could have a significant impact on the occupational risk prevention practices of construction site employees. This is a useful finding, given that all employees in France systematically and regularly have such medical visits. Our results highlight a potentially important lever for the prevention of occupational risks in the construction field in the future through the improvement of employee competence.

### Other Uses of Virtual Reality in the Health Field

Eiris et al [22] sought to validate safety training using 360-degree augmented reality panoramas. Their study showed the interest in the use of this method in the identification and recognition of hazards on construction sites. However, the rate of hazard identification was quite low, as only 30% of the hazards were identified by the participants. They explained this by the fact that their population was composed of students specializing in construction management (n=30) and were not building and construction professionals. They also emphasized the constructive comments concerning the ease of use of the platform, feedback that we also had in our study using VR. In our study, we did not analyze the responses to the questions asked during the VR training, as this did not correspond to our research question.

Nykänen et al [10] evaluated both the effectiveness of an immersive VR-based safety training program and a participatory human factors safety training program. The study was conducted with 119 employees working on 8 construction sites in Finland. The employees evaluated the training with questionnaires at the start, immediately after the intervention, and at a 1-month follow-up. They considered VR to be a serious tool for improving prevention skills and found that it motivated them to apply prevention rules more than after safety training based on passive learning methods. This study was conducted only with employees of medium-sized and large companies.

Simeonov et al [23] investigated the value of reducing mechanical vibration of support structures used as walking or working surfaces when performing construction tasks at height (falls from height account for one-third of fatal accidents in construction). Employees (n=12) used instrument-carrying gel insoles connected to a VR system to test sensory perception of the feet. The study did not show any effectiveness for this technology in 2008, but given the evolution of VR technologies, it is possible that the results would be different today.

We also found studies that assessed the use of VR as a prevention and training tool in fields other than construction.

The mining industry is a field in which the risk of serious accidents and fatalities is very high. Filigenzi et al [24] highlighted the value of using VR to train surface and underground mine employees and rescue personnel in hazard recognition and evacuation routes and procedures. This study, carried out in 2000, was innovative, demonstrated possibilities, and generated interest in extending such an approach to other fields of high-risk activity, such as construction, agriculture, and the oil industry.

In the logistics field, the use of handling equipment is responsible for a large number of occupational accidents, in particular to third parties. Choi et al [25] focused on forklift drivers, conducting a study with 20 students at Hong Kong Polytechnic University specializing in construction engineering. Their goal was to investigate how a forklift driver's situational awareness of others around him can be influenced by the type of subtasks he performs. A VR environment was used as the experimental environment in which participants performed a series of subtasks, such as driving, turning, reversing, loading, and unloading: the more concentration that was required for the tasks, the higher the risk of an accident. The authors concluded that it would be beneficial to not only use additional safety devices (such as person detection devices) but also have more detailed safety training, making VR meaningful.

In the area of electrical risk training, in 2015, Zhao and Lucas [26] reported that human error was responsible for approximately 50% of all electrical-related fatalities in multiple industries in the United States. They hypothesized that effective employee safety training programs, including VR, would be the most direct approach to mitigate such errors. Their study showed the success of using VR, highlighting training that effectively visualizes invisible risks without endangering employees. Such training increases awareness of the risk and trains employees to use the necessary protective equipment.

In the health care field, VR interventions appear to be an effective tool to boost the intention to be vaccinated [27-29].

The results of our study, as well as those of others in various fields, show that VR training tools hold great potential and should be further developed to improve the prevention of occupational risks, particularly in the construction field.

### Strengths and Limitations

One of the strengths of our study is that it was conducted with a large population and 2 groups who were similar in terms of gender. In addition, the completion rate was 100% due to the use of a short and acceptable questionnaire.

However, the study population was mainly composed of men, which did not allow us to obtain data on the female population in the construction field. Women are not as well represented as men in the national population of construction employees. In addition, this intervention was intended only for certain construction jobs, mainly on construction sites, where women are much less present. The female population is mainly present in the administrative field of construction and public works companies and is therefore not subject to the same occupational risks.

Employees in the MV+VR group were younger than those in the MV group, which is similar to the overall population of construction employees in France. This result was expected, given the appetite of the younger generation for new technologies, such as VR. This age difference suggests that, if this tool is deployed on a larger scale, the older portion of the construction employee population might not benefit from it, as they may not want to use it.

The employees in the MV+VR group were also more often from small companies, which can be explained by the fact that they were the target population for the occupational health service. It is possible that this influenced our results, as larger companies have more resources for prevention. The employees of larger companies might therefore find this training less useful, but we believe that this does not affect the interpretation of our results.

It should also be noted that the use of VR is already a common practice in occupational health services and that our study did not change these practices, apart from the addition of the questionnaire. We therefore believe that our intervention did not bias our results.

On the other hand, we excluded individuals with the least mastery of the French language from our study. Individuals in this group are among those most at risk of having an accident at work due to the language barrier, in particular because of difficulties in understanding safety instructions. This does not call into question the validity of our results but highlights this group’s limited access to prevention through this tool. A translated version could be envisaged.

All the employees who participated were only seen once by the SMIBTP. It was therefore not possible to evaluate the impact of repeating these VR training sessions. Similarly, the design of the study did not allow an evaluation of the impact of this training at a later date. This was a major limitation of our study. Although these results are encouraging, other studies are needed to evaluate the long-term impact of VR training on the knowledge and perception of personal effectiveness in preventing occupational hazards. Longer-term studies are also needed to study the tool’s impact in terms of reducing the occurrence of occupational accidents.

In the context of our study, no data were collected that could be used to identify employees. Our objective was to reproduce, as closely as possible, the real-life conditions of using the tool, and we knew that collecting identification data could have significantly reduced participation in our study. If we were to carry out an evaluation at a later time point, it would logically be conducted under the normal conditions of periodic medical visits in occupational health services and therefore completed within 2 years to 5 years following our study. We would ask the employees coming for a visit whether they had already received training via VR. If so, we would request that they complete a questionnaire.

Further studies will be needed to assess the acceptability of VR. Indeed, one of the classic side effects of VR is motion sickness, and some VR sessions had to be interrupted because of symptoms such as nausea [30]. VR can also alter sensorimotor and perceptual abilities, with effects that can last several hours after exposure, and cause visual fatigue and headaches [31].

The routine use of VR during medical visits by occupational health services could have an impact on occupational risk prevention in the construction field. It could be a tool of major importance, given its accessibility, but its long-term impact and accessibility need to be assessed.

## Abbreviations

MV: medical visit
SMIBTP: Services Médicaux Interentreprises Bâtiment Travaux Publics
VR: virtual reality
